# Evidence for Gender-Specific Bone Loss Mechanisms in Periprosthetic Osteolysis

**DOI:** 10.3390/jcm9010053

**Published:** 2019-12-24

**Authors:** Renee T. Ormsby, Lucian B. Solomon, Roumen Stamenkov, David M. Findlay, Gerald J. Atkins

**Affiliations:** 1 Biomedical Orthopaedic Research Group, Centre for Orthopaedic & Trauma Research, The University of Adelaide, Adelaide, SA 5000, Australia; renee.ormsby@adelaide.edu.au; 2 Centre for Orthopaedic & Trauma Research, The University of Adelaide, Adelaide, SA 5000, Australia; lucian.solomon@adelaide.edu.au (L.B.S.); david.findlay@adelaide.edu.au (D.M.F.); 3 Department of Orthopaedics & Trauma, Royal Adelaide Hospital, Adelaide, SA 5000, Australia; roumen.stamenkov@sa.gov.au

**Keywords:** periprosthetic osteolysis, aseptic loosening, total hip replacement, osteocyte, osteocytic osteolysis, wear particles

## Abstract

Osteolysis adjacent to total hip replacement (THR) prostheses is a major cause of their eventual failure. Periprosthetic osteolysis is associated with the production of bioactive particles, produced by the wear of articulating prosthesis surfaces. Wear particles invade the periprosthetic tissue, inducing inflammation and bone resorption. Previous studies have shown that osteocytes, the most numerous cell type in mineralised bone, can respond to wear particles of multiple orthopaedic material types. Osteocytes play important roles in bone resorption, regulating bone resorption by osteoclasts and directly through osteocytic osteolysis, also known as perilacunar remodelling. In this study, we perform a histological analysis of bone biopsies obtained from cohorts of male and female patients undergoing either primary THR surgery or revision THR surgery for aseptic loosening. The osteocyte lacunae area (Ot.Lac.Ar) and percentage lacunar area/bone area (%Ot.Lac.Ar/B.Ar) were significantly larger overall in revision THR bone than bone from similar sites in primary THR. Analysis by patient gender showed that increased Ot.Lac.Ar, indicative of increased perilacunar remodelling, was restricted to female revision samples. No significant differences in osteoclast parameters were detectable between the cohorts. These findings suggest previously unrecognised gender-specific mechanisms of bone loss in orthopaedic wear particle-induced osteolysis in humans.

## 1. Introduction

Osteoarthritis is a common joint disorder that leads to total hip replacement (THR) surgery when non-operative treatments fail. THR alleviates pain and restores mobility to the joint, however, failure of the implant can occur, commonly due to loosening [1]. Aseptic osteolytic lesions have been identified as a main cause of loosening in implants [2,3,4,5,6,7]. Osteolytic lesions are visualised as radiolucent areas within the bone architecture, more commonly seen in the cancellous bone above the acetabular component of an implant [8]. The lesions become devoid of bone and infiltrated with granulomatous tissue that contains multiple cell types including macrophages, fibroblasts, osteoclasts and inflammatory cells [6,9]. 

The production of bioactive wear particles has been identified as a main cause of osteolysis [10]. Polyethylene (PE) and metal particles formed by the abrasive wear of bearing surfaces and modular component fretting have been shown to infiltrate the periprosthetic tissue [11], be highly bioactive and cause adverse tissue reactions [12]. Both metal and PE particles have been shown to induce biological reactions, including promoting both bone resorption and inflammatory pathways, whilst inhibiting bone formation [13,14,15,16]. 

The analysis of bone biopsies from patients, who have undergone revision surgery, has led to the identification of changes in the bone microenvironment caused by wear particles, including the upregulation of key inflammatory genes and osteoclastic markers, as well as the detection of wear particles within the mineralised bone surrounding implants [17]. Data are emerging that osteocytes, the most abundant cell type in the bone, play active roles in periprosthetic osteolysis [18,19]. Through the lacunocanalicular network, osteocytes can sense mechanical strain and biochemical signals, and in response, influence both bone formation and resorption by sending regulatory stimuli to surface osteoclasts, osteoblasts and bone lining cells [20,21]. In females, the osteocyte plays an important physiologic role to release calcium from the bone matrix during lactation, through the process of osteocytic osteolysis, also known as perilacunar remodelling [22]. This occurs through the osteocytic production of key bone degrading enzymes, including matrix metalloproteinases (MMP’s) [23], cathepsin K [22] and carbonic anhydrase II [24], which degrade both the organic and inorganic components of the bone mineral matrix, increasing the lacunar area and releasing calcium into the circulation [25]. This process may also be regulated by calcitonin, traditionally thought to be a regulator of osteoclastic bone resorption [26]. We have previously identified that osteocytes also respond to wear particles of multiple orthopaedic material types, including both conventional and cross-linked UHMWPE, Ti6Al4V and CoCrMo, by the upregulation of both pro-osteoclastic and osteocytic osteolysis pathways [19,27]. 

In a previous study, we showed histological evidence of osteocytic osteolysis with increased osteocyte lacunar area in a case series of patients undergoing revision THR surgery for aseptic loosening with confirmed radiographic evidence of periprosthetic osteolysis [19]. In the current study, we examine osteocyte histology in a larger cohort of patients, undergoing revision for aseptic loosening, with confirmed radiographic evidence of periprosthetic osteolysis, and compare it to a gender and age-matched cohort of patients undergoing primary THR. We confirm here that osteocytic osteolysis is a feature of periprosthetic osteolysis, however this process appears to occur predominantly in females, suggesting for the first time that cellular mechanisms of pathological bone loss may be gender specific. 

## 2. Experimental Section

### 2.1. Ethical Statement

Patients were recruited into this study with informed written consent and with the ethics approval by the Human Research Ethics Committees of the Royal Adelaide Hospital and the University of Adelaide (RAH Approval No. 130114 and 140216a).

### 2.2. Patient Demographics

Twenty patients undergoing primary THR for osteoarthritis and twenty-one patients undergoing revision THR for aseptic loosening associated with radiographic evidence of periprosthetic osteolysis were recruited at the Royal Adelaide Hospital. All revision patients had a pre-operative CT scan for planning purposes, patient demographics are shown in Table 1. Due to the advanced age of a revision THR patient, the primary THR patients were included within the age range, eliminating any significant difference between the cohort ages. The patient number also included comparable numbers of males and females. Revision THR patient implant details, shown in Table 2, show a varying selection of implant manufacturer and metal alloy type. All prostheses contained a conventional UHMWPE liner, however prosthetic details for two patients are unknown. 

### 2.3. Osteocyte and Osteoclast Histomorphometric Analysis of Human Bone Biopsies

Intraoperative trephine or curette biopsies ranging in size from 4 to 20 mm in length and 3 to 10 mm in breadth were taken from the periacetabular bone prior to reaming for insertion of the acetabular component, in the patients undergoing primary THR, and after removal of the failed implant and the granulomatous tissue, in the patients undergoing revision THR. The biopsies were fixed in 10% neutral buffered formalin for 48 hours and then decalcified (10% EDTA/1% paraformaldehyde) for 2 weeks. Sections (5 µm) were cut and stained with toluidine blue and TRAP, as described previously [19], and imaged using NanoZoomer (Hamamatsu Photonics, Shizuoka, Japan) at 40x magnification. Osteocyte lacunae perimeters and bone perimeters were manually traced for each section, using a Bamboo Pen and Touch (Wacom, Kazo, Saitama, Japan) [19], and quantified using the Freehand region measurement tool in the NanoZoomer software (Hamamatsu Photonics, Shizuoka, Japan) to generate the histomorphometric measurements of total bone area (B.Ar), mean osteocyte lacunar area (Ot.Lac.Ar), percent osteocyte lacunar number/bone area (N.Ot./B.Ar (%)), and percent osteocyte lacunar area/bone area (Ot.Lac.Ar/B.Ar (%)) and were quantified using Image J software (U. S. National Institutes of Health, Bethesda, MD, USA). For osteoclast measurements, the osteoclast number/bone area (N.OC./B.Ar (%)) and osteoclast area/bone area (OC.Ar/B.Ar (%)) area were similarly measured by manual tracing of TRAP-stained sections [19]. Measurements were performed on at least two sections per patient/stain, and the entire section was examined in all cases. 

### 2.4. Statistical Analysis

Statistical differences between the osteocyte parameters (normally distributed) were assessed using Student’s t-tests (GraphPad software v7.02). Statistical differences between N.OC./B.Ar (%) and OC.Ar/B.Ar (%) were assessed using the Mann–Whitney U non-parametric test. Significant differences were accepted for *p* values < 0.05. 

## 3. Results

### 3.1. Radiographic Evidence of Osteolysis in Patients Undergoing Revision THR

**Figure 1:**
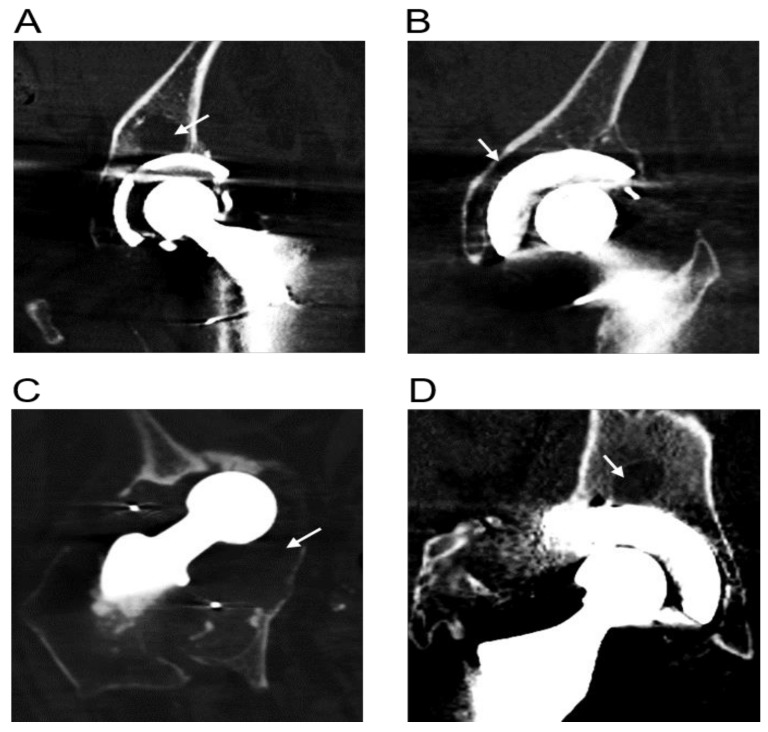
Representative radiographs of four revision patients with osteolytic lesions (white arrows). Corresponding patient and implant details are listed in Table 2 and are as follows: (**A**) Patient 1, (**B**) Patient 2, (**C**) Patient 8 and (**D**) Patient 14.

Pre-operative CT scans obtained for all 21 revision THR patients showed evidence of osteolysis. Representative CT images showing examples of osteolytic zones are shown in Figure 1.

### 3.2. Analysis of Human Bone Biopsies from Patients Undergoing Primary THR and Revision THR Surgery

**Figure 2:**
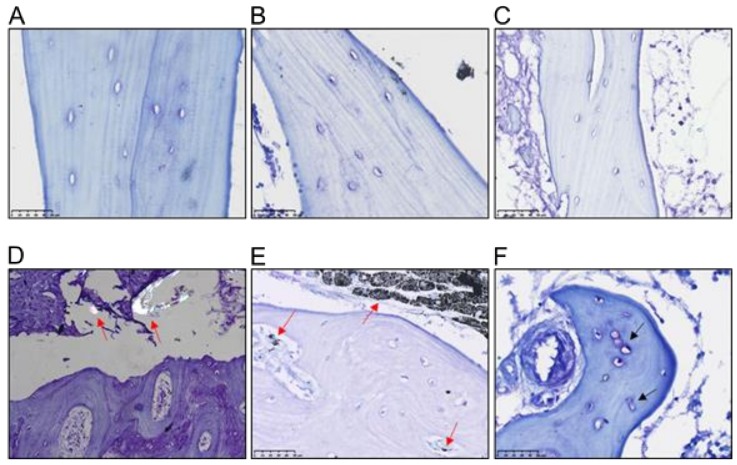
Bone biopsies were obtained from Primary THR (**A**–**C**) and Revision THR surgeries (**D**–**F**) and stained with toluidine blue. Images depict sections from three individual patients per group. Revision THR biopsies shown enlarged osteocyte lacunae with the presence of PE particles (2D), as well as metal particles and granuloma in 2E (red arrows). Evidence of osteocyte coalescence is discernible in 2F (black arrows).

Representative images of bone morphometry of primary THR and revision THR biopsies are shown in Figure 2A–C,D–F, respectively. Histological analysis of the bone biopsies showed metal wear particles evident within granulomatous tissue adjacent to the bone (Figure 2E). Particles of either metal or UHMWPE were observed by microscopy in 17 of the 21 biopsies taken from the patients in the revision cohort; in seven patients’ samples both particle types were detected (five female, two male). Metal particles were detected in eight of the female biopsies and in three male biopsies (*n* = 11), evidence of UHMWPE particles were detected in six female biopsies and in seven male biopsies (*n* = 13). Metal particles were additionally identified in Haversian canals (Figure 2E). Evidence of osteocyte lacunae coalescence was also observed (Figure 2F).

### 3.3. Osteocyte Characteristics of Primary and Revision THR Ccohorts

**Figure 3:**
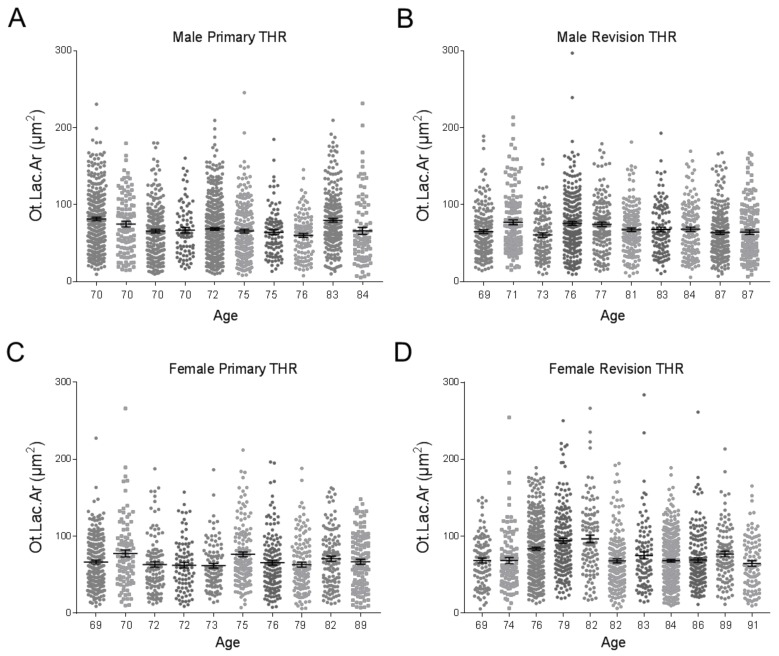
Column scatter plots of quantified osteocyte lacunar areas (Ot.Lac.Ar (µm^2^)) for each patient biopsy with corresponding patient age: (**A**) male Primary THR patient biopsies; (**B**) male Revision THR patient biopsies; (**C**) female Primary THR patient biopsies and (**D**) female Revision THR biopsies. THR: total hip replacement.

**Figure 4:**
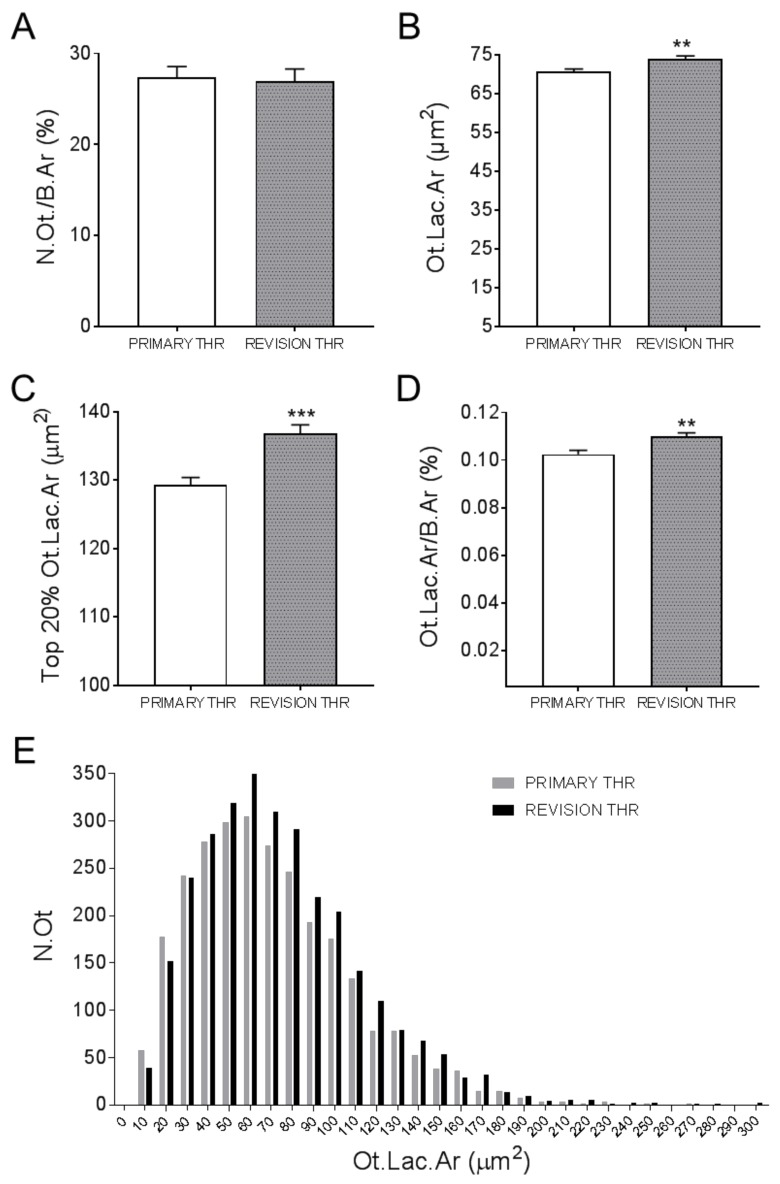
Osteocyte characteristics for human Primary THR compared to Revision THR biopsies: (**A**) osteocyte number per bone area (N.Ot./B.Ar (%)); (**B**) average osteocyte lacunar area (Ot.Lac.Ar (µm^2^)); (**C**) the top 20% lacunar areas (Top 20% Ot.Lac.Ar (µm^2^)); (**D**) the percentage lacunar area per total bone area (Ot.Lac.Ar/B.Ar (%)) and (**E**) histogram of the osteocyte lacunar size distribution (Ot.Lac.Ar (µm^2^)), ranging from 60 to 300 µm^2^. Data shown are means ± standard errors of the mean (SEM). Significant differences are denoted by **p < 0.01, ***p < 0.001.

Osteocyte lacunar areas (Ot.Lac.Ar) were measured for each patient. These are shown for males (Figure 3A–B) and females (Figure 3C–D), in the primary or revision cohorts, respectively. Quantification of the percentage of osteocytes per total bone area revealed no significant difference between the primary and revision THR cohorts, irrespective of gender (Figure 4A). However, there was a significant increase in the mean Ot.Lac.Ar in the revision compared to the primary THR cohort (Figure 4B). Due to the 3D ellipsoid shape of an osteocyte lacunae, the measurement of the 20% largest osteocyte lacunar area (Top 20% Ot.Lac.Ar) was considered, which corresponds to the likely mid-cross sections of lacunae in a section [28]. This measure was also significantly increased in the revision biopsies compared to the primary THR biopsies (Figure 4C). The percentage osteocyte lacunar area per total bone area (Ot.Lac.Ar/B.Ar (%)) was also significantly increased in the biopsies from revision THR (Figure 4D). Examination of size distribution revealed a greater number of large lacunae (60–300 µm^2^) in biopsies from the revision THR group compared to the primary THR group (Figure 4E). 

### 3.4. Osteocyte Characteristics of Female THR and Male THR Bone Biopsies

**Figure 5:**
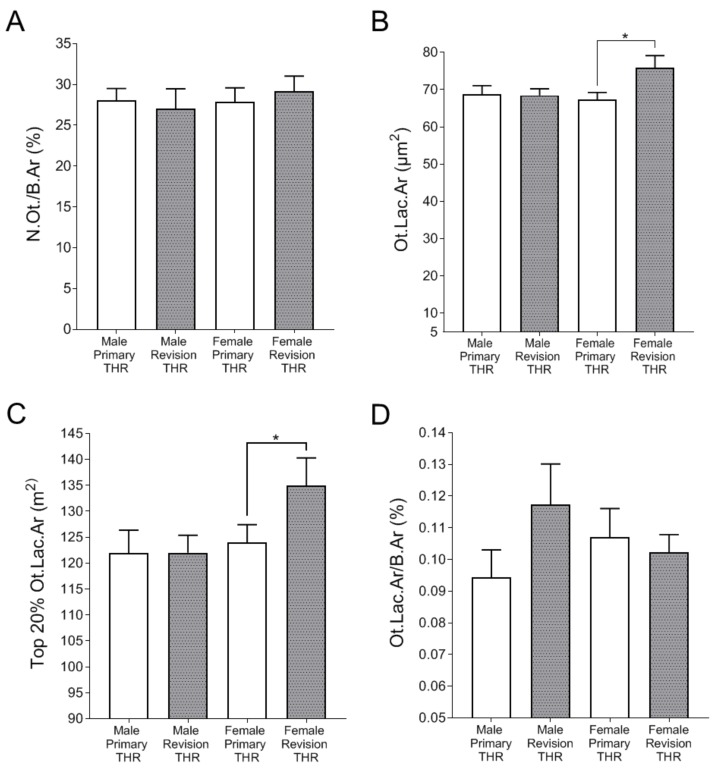
Gender-specific analysis of osteocyte lacunar properties: (**A**) osteocyte number per bone area (N.Ot./B.Ar (%)) for Male Primary THR, Male Revision THR, Female Primary THR and Female Revision THR biopsies; (**B**) average lacunar area (Ot.Lac.Ar (µm^2^)); (**C**) the top 20% lacunar area (top 20% Ot.Lac.Ar (µm^2^)) for each cohort; (**D**) The percentage lacunar area per total bone area in all cohorts (Ot.Lac.Ar/B.Ar (%)). Data shown are means ± standard error of the mean (SEM). Significant differences are denoted by *p < 0.05.

A further analysis was conducted on the basis of patient gender. There was no difference between groups in terms of number of osteocytes per bone area (Figure 5A). The analysis of the average lacunar area revealed significantly larger lacunae in the female revision cohort, compared to female primary THR cohort (Figure 5B). Similarly, the top 20% Ot.Lac.Ar measure was significantly larger in the female revision THR samples compared to the female primary THR samples (Figure 5C). In contrast, there were no differences in the corresponding Ot.Lac.Ar measurements between male revision and primary THR cohorts (Figure 5B–C). However, the percentage lacunar area per total bone area was not significantly different between cohorts when analysed on the basis of gender (Figure 5D). 

### 3.5. Osteoclast Analysis of Primary and Revision THR Bone Biopsies

**Figure 6:**
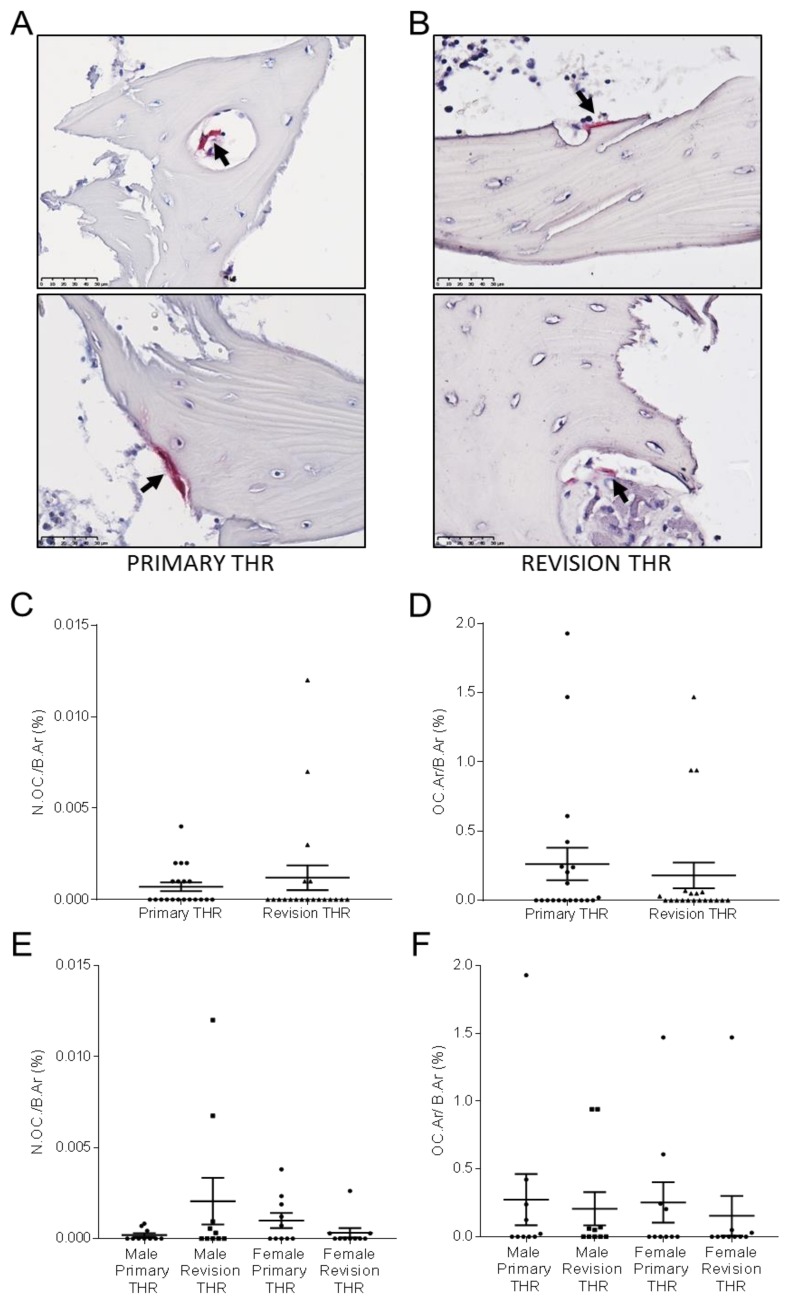
Bone biopsies were obtained from Primary THR (**A**) and Revision THR surgeries (**B**) and stained with TRAP. Images depict sections from two individual patients per group, TRAP positive osteoclasts were observed (black arrow) in both Primary THR and Revision THR cohorts. Osteoclast characteristics for human Primary THR compared to Revision THR biopsies: (**C**) the osteoclast number per bone area (N.OC./B.Ar (%)) for primary THR compared to revision THR cohort; (**D**) osteoclast area per bone area (OC.Ar/B.Ar (%)) for primary THR compared to revision THR cohort; (**E**) osteoclast number per bone area (N.OC./B.Ar (%)) for Male Primary THR, Male Revision THR, Female Primary THR and Female Revision THR cohorts; (**F**) osteoclast area per bone area (OC.Ar/B.Ar (%)) for Male Primary THR, Male Revision THR, Female Primary THR and Female Revision THR cohorts. Data shown are means ± standard error of the mean (SEM).

Bone sections were stained for TRAP-positive osteoclasts; representative images for the primary and revision cohorts are shown in Figure 6A–B. The presence of osteoclasts in these samples was a relatively rare event, demonstrated by the very low values for %N.OC./B.Ar., in general less than 0.005%, being approximately 6000-fold lower than the corresponding percentage for osteocytes (N.Ot./B.Ar.(%)), which, as shown in Figure 5A, was between 25–30%. There was no difference between the overall primary and revision THR cohorts in terms of N.OC./B.Ar (%) (Figure 6C). Likewise, there was no gender-specific difference in this measure (Figure 6E). There was however, a significantly greater variance in N.OC./B.Ar (%) between male and female revision samples (F-test, *p* < 0.001), suggesting dysregulated osteoclastogenesis in the male revision samples. The percentage osteoclast area per total bone area (OC.Ar/B.Ar (%)) was also quantified, and no significant difference was observed between the primary and revision cohorts (Figure 6D), or between the gender cohorts (Figure 6F). 

## 4. Discussion

There is an emerging role for osteocytes in the development of periprosthetic osteolysis [5,19]. In this study, we analysed bone biopsies taken from sites adjacent to osteolysis in patients undergoing revision THR for aseptic loosening. Microscopic examination of bone biopsies confirmed the presence of particulate material in 17/21 revision THR patients, linking the production of wear particles and the development of osteolysis in these patients [10,29,30]. Histological analysis revealed significantly increased mean osteocyte lacunar size in the revision THR bone when measurements from both male and female biopsies were combined, compared to those in cases of primary THR, consistent with our previous report [19]. Additionally, there was an overall substantial increase in the % lacunar area per total bone area in the revision THR biopsies compared to primary samples. 

A significant finding of this study is that evidence for increased osteocytic osteolysis in revision THR was restricted to the female samples, and differences in osteocyte lacunar size were not observed in the corresponding male samples. This suggests that the female osteocyte response to either wear particles or another catabolic influence in this disease, may be preferentially directed towards osteocytic osteolysis. In comparison, the percentage osteocyte lacunar area as a function of the total bone area was not altered in female bone, and the observed increase in this measure in revision samples appeared restricted to males, although this was not significant in the gender-specific analysis. Analysis of osteoclast parameters of both primary and revision cohorts showed no significant difference in osteoclast number per total bone area or percentage osteoclast area per bone area. Analysis of the gender cohorts also showed no significant differences; however, a qualitatively greater number of osteoclasts were observed in the male revision THR samples compared to female revision THR. Males have a reported increased risk of developing osteolysis [31], possibly due to their increased physical activity [32], which could contribute to an increase in the production of osteolysis-associated wear particles. 

The reason(s) for the difference in osteocyte responses between the genders is not clear. However, as the women in the cohorts examined were all postmenopausal, this suggests that hormonal changes associated with post-menopause may play a role. A major physiological role of osteocytic osteolysis is the release of calcium from the perilacunar matrix during lactation, as previously described in mice [22,23,26,33]. It is possible that this process is linked to the action of the pituitary hormone prolactin, which together with the decrease in oestrogen levels, is known to be essential for the onset of lactation [34]. Additionally, it has been shown that prolactin receptors have been identified in osteoblasts, and activation of these receptors inhibits osteoblast activity and has also been shown to induce the expression of RANKL relative to its inhibitor OPG [35]. Therefore, it seems plausible that changes in prolactin levels may stimulate osteocytic responses that have not been previously described. It has also been shown that increased levels of other pituitary hormones play important roles in regulating bone mass, including follicle stimulating hormone, which has been shown to regulate osteoclastic bone resorption in mice and, importantly, correlates with increased markers of bone resorption in perimenopausal women [36]. Thus, the hormonal changes that occur during menopause may predispose female osteocytes to induce perilacunar remodelling in general, contributing to the loss of bone [37]. In the specific case of periprosthetic osteolysis, these hormones may also interact with the effects of particles to produce a catabolic phenotype in osteocytes, however a mechanism for this is currently unknown. Furthermore, we observed evidence of lacunae enlargement to the extent of coalescence, implying that in females, osteocytic osteolysis can potentially contribute to the macroscopic lesions’ characteristic of this pathology.

Multiple particle types have been shown to induce the expression of osteocytic osteolysis mediators [19,27]. Therefore, these findings further support a key role for osteocytic bone resorption in wear particle disease. The revised patient prostheses within this cohort consisted of a variety of implant types with varying alloys, including Ti6Al4V and vitallium (CoCrMo). Additionally, all but one patient’s prosthesis in this study included a conventional UHMWPE liner. Investigation of bone biopsies obtained from this cohort showed histological evidence of metal wear particle invasion into the bone in 11/21 cases, identified primarily in the patients with a cobalt chrome alloy implant. Furthermore, histological analysis under polarised light also showed the presence of birefringent UHMWPE particles in 13/21 biopsies, as previously reported [19]. Interestingly, 7/21 patients’ biopsies showed evidence of both metal and UHMWPE particles. Metal particles were visible in the Haversian canals, as well as in the granuloma surrounding the bone. The close proximity of wear particles to bone would enable direct contact with osteocytes. We have previously reported that human osteocyte-like cells were capable of physically attaching and engulfing UHMWPE particles [18]. More recently, it was demonstrated that human osteocytes internalise viable *Staphylococcus aureus* bacteria [38], which at 500–700 nm are similar in size to bioactive wear particles. We cannot rule out that all revision patient biopsies in this study contained sub-micron sized wear particles that we could not detect.

There are several limitations to this study. The patients recruited were from two separate cohorts, primary THR and revision THR for loosening, and the changes in osteocyte measures may have been pre-existing. Alternatively, the surgical implantation of a prosthesis into the joint space in the revision THR group could have potentially altered the intrinsic characteristics of the bone. During the procedure, surgical reaming removes the cortical shell and exposes the underlying trabecular bone. This alone may lead to inflammatory effects that could affect osteocyte lacunar area and porosity, although we are not aware of published data regarding this. However, we found a clear difference between males and females suggesting that these possibilities did not contribute to the effects seen. This study is also cross-sectional; a longitudinal study would be preferable, however the unpredictable and long-term nature of this pathology and the increased use of wear-resistant materials, such as XLPE, would make this difficult. Furthermore, we have not identified a basis for the gender-specific effect. While none of the patients in this study were reported as having clinical signs of metallosis or metal hypersensitivity, factors such as BMI, drug status, levels of physical activity and underlying comorbidities were not taken into account. Conditions including diabetes mellitus [39], a history of smoking [40] and obesity [41] have all been shown to affect bone health, and thus could potentially contribute to the changes observed. A larger cohort of patients with sufficient statistical power to allow multivariate analysis of all potentially contributing factors would help elucidate the influence of gender on the possible mechanisms of bone loss. Another potential confounder of this and future studies is the wide variety of hip implants and materials used, as evident in Table 2, and therefore the multiple possible types and combinations of wear particles released. A larger cohort of patients would likely provide further insight into which particles and combinations of particles are most clinically relevant in this context 

## 5. Conclusions

In conclusion, this study provides evidence for different modes of bone loss between male and female patients with periprosthetic osteolysis associated with aseptic loosening. Wear particle-associated bone loss consists of both osteoclastic and osteocytic bone resorption, with osteocytes playing key roles in both pathways. However, our current findings suggest that the latter process may be specific to females. It is conceivable that female osteocytes are pre-programmed to undergo osteocytic osteolysis under conditions of rapid or pathologic bone loss, as this mechanism is important for the release of calcium during lactation. Alternatively, the altered hormonal status of women through post-menopause may drive this process. Further investigation is required to elucidate the mechanisms of gender specificity of bone loss and the conditions under which this occurs. In summary, osteocytes may contribute to the development of osteolysis through stimulation of resorptive pathways in response to wear particles, contributing to the aseptic loosening of implants, in a gender-specific manner.

## Figures and Tables

**Table 1:** Patient demographics for primary and revision cohorts. THR: Total hip replacement.

	Age Range	Mean Age	Number Per Group
Primary THR	69–89	74	20 (M 10, F 10)
Revision THR	69–91	80	21 (M 10, F 11)
Male Primary THR	70–83	75	10
Male Revision THR	69–87	79	10
Female Primary THR	69–89	75	10
Female Revision THR	69–91	80	11

**Table 2:** Revision THR patient implant details. UHMWPE: Ultra-high molecular weight polyethylene; XLPE: Cross-linked polyethylene; CPT: collarless, polished, double taper; PCA: Porous Coated Anatomic; HGP: Harris-Galante Porous; CLS: Cemented Locking System.

Patient	Sex	Age	Acetabular Cup Name	Cup Material	Liner	Stem Name	Stem Material	Particle Type
1	F	69	Trilogy	Titanium	UHMWPE	CPT©	Co-Cr Alloy	Metal
2	F	74	PCA	Vitallium	UHMWPE	PCA	Vitallium	Not detected
3	F	76	HGP	Titanium	UHMWPE	Zimmer Anatomic II	Titanium	Both
4	F	79	Exeter	Steel	UHMWPE	Exeter	Steel	Metal
5	F	82	HGP	Titanium	UHMWPE	HGP	Titanium	Both
6	F	82	Unknown	Unknown	UHMWPE	Unknown	Unknown	Both
7	F	83	Exeter	All-Poly Cup	UHMWPE	Exeter polished monoblock	Stainless Steel	PE
8	F	84	None	None	None	Exeter Hemiarthroplasty	Stainless Steel	Metal
9	F	86	Trilogy	Titanium	XLPE	MULLER	Stainless steel and Titanium	Both
10	F	89	MULLER	All-Poly Cup	UHMWPE	MULLER	Co-Cr-Mo-Ni	Both
11	F	91	Unknown	Unknown	Unknown	Unknown	Unknown	Not detected
12	M	69	Howmedica All-Poly Cup	All-Poly Cup	UHMWPE	Howmedica Osteonics ODC	Co-Cr Alloy	Metal
13	M	71	Charnley	All-Poly Cup	UHMWPE	Unknown	Unknown	Not detected
14	M	73	PCA	Vitallium	UHMWPE	PCA	Vitallium	Both
15	M	76	Biomet	Titanium	UHMWPE	Unknown	Unknown	PE
16	M	77	PCA	Vitallium	UHMWPE	PCA	Vitallium	Both
17	M	81	Charnley	All-Poly Cup	UHMWPE	Charnley	Stainless Steel	PE
18	M	83	Trilogy	Unknown	UHMWPE	CPT	Co-Cr Alloy	Not detected
19	M	84	CLS	Titanium	UHMWPE	CLS	Titanium	PE
20	M	87	Meridian	Vitallium	UHMWPE	Vitaloc	Vitallium	PE
21	M	87	Unknown	Unknown	Unknown	Unknown	Unknown	PE
